# Subjective Well-being and Partnership Dynamics: Are Same-Sex Relationships Different?

**DOI:** 10.1007/s13524-018-0725-0

**Published:** 2018-11-01

**Authors:** Shuai Chen, Jan C. van Ours

**Affiliations:** 1 Department of Economics and CentER, Tilburg University, P.O. Box 90153, Room P2.115, 5000 LE Tilburg, the Netherlands; 2 Erasmus School of Economics, Postbus 1738, Room H12-10, 3000 DR Rotterdam, the Netherlands; 3 Department of Economics, University of Melbourne, Melbourne, Australia; 4 Tinbergen Institute, Amsterdam, the Netherlands; 5 EHERO, Rotterdam, the Netherlands; 6 CEPR, London, UK

**Keywords:** Subjective well-being, Happiness, Marriage, Cohabitation, Same-sex relationships

## Abstract

We analyze Dutch panel data to investigate whether partnership has a causal effect on subjective well-being. As in previous studies, we find that, on average, being in a partnership improves well-being. Well-being gains of marriage are larger than those of cohabitation. The well-being effects of partnership formation and disruption are symmetric. We also find that marriage improves well-being for both younger and older cohorts, whereas cohabitation benefits only the younger cohort. Our main contribution to the literature is on well-being effects of same-sex partnerships. We find that these effects are homogeneous to sexual orientation. Gender differences exist in the well-being effects of same-sex partnerships: females are happier cohabiting, whereas marriage has a stronger well-being effect on males.

**Electronic supplementary material:**

The online version of this article (10.1007/s13524-018-0725-0) contains supplementary material, which is available to authorized users.

## Introduction

In the past decades, numerous studies in economics, sociology, and demography emerged on the relationship between partnership and well-being or happiness.^1^ This literature has predominantly asserted a positive association between marriage and well-being (Carr and Springer 2010; Diener and Suh 1997; Gove and Shin 1989; Kalmijn 2017; Umberson and Karas Montez 2010; Waite and Gallagher 2000). Recently, a few studies examined whether such a positive relationship exists between cohabitation and well-being, finding mixed results (Brown et al. 2005; Hansen et al. 2007; Kamp Dush 2013; Kohn and Averett 2014a; Musick and Bumpass 2012; Soons and Kalmijn 2009; Soons et al. 2009; Wright and Brown 2017).

The positive association between partnership and well-being could originate from a causal effect of partnership on happiness. However, the positive association could also be due to selection: happier individuals are more likely to enter a partnership (Johnson and Wu 2002; Kalmijn 2017; Kim and McKenry 2002; Sandberg-Thoma and Kamp Dush 2014; Stutzer and Frey 2006; Waldron et al. 1996; Wilson and Oswald 2005).^2^ Four nonexclusive explanations can be offered for the causal effect. First, partnered individuals may gain from *production complementarities*—that is, specialization and division of labor (Becker 1974, 1981; Stutzer and Frey 2006). Second, partnership may offer *consumption and investment complementarities* (Lundberg and Pollak 2015; Stevenson and Wolfers 2007). Couples may benefit from economies of scale by pooling resources, jointly consuming public goods and investing in children, and sharing leisure activities (Killewald 2013; Waite and Gallagher 2000). Third, a partnership may strengthen and expand social relationships. Partnered individuals not only receive intimacy, commitment, and care from their partner but also obtain material and emotional support from the family, relatives, and friends of their partner (Kamp Dush and Amato 2005; Ross 1995). Finally, a partnership may introduce social control and mutual supervision salutary to the couple’s well-being. The norms in a partnership and the daily supervision by the partner reduce possible risky behavior (Duncan et al. 2006; Fleming et al. 2010; Monden et al. 2003; Umberson 1992).

We investigate the well-being effects of partnership dynamics in the Netherlands, which has witnessed notable demographic changes in the past decades. In terms of partnership formation, cohabitation has become more popular at the expense of marriage. For example, by age 30, 34 % of women born in the 1950s had been or were still cohabiting, and 78 % had been or were still married. Among women born in the 1970s, these percentages switched by age 30 to 69 % for cohabitation and 45 % for marriage. In 1998, there were approximately 3.4 million married couples, 0.6 million cohabiting households, and 2.2 million single households. In 2016, the number of married couples decreased to 3.3 million, and the numbers of cohabiting couples and single households increased to 1.0 and 2.9 million, respectively. Furthermore, fewer cohabiting couples made a transition into marriage. For instance, for cohabiting women aged 20–24, the probability of being married within three years after the start of cohabitation clearly dropped. For those starting to cohabit in 1970–1974, this probability was 58 %; for those starting to cohabit in 1980–1984, the probability reduced to 37 %; and for the 1990–1994 cohort, the probability further fell to 27 %. In the meantime, divorce rates have risen. In 1970, approximately 0.3 % of all marriages dissolved; in 2014, this rate was approximately 1 % (Statistics Netherlands n.d.).

Our study exploits panel data on partnerships and subjective well-being collected in the Netherlands over the period 2008–2013. Our data allow us to distinguish between marriage and cohabitation and between different-sex and same-sex relationships. Couples may invest different levels of tangible and intangible capital (Michael 2004) in marriage and cohabitation (Nock 1995; Stanley et al. 2004), and thus the subjective well-being derived from cohabitation and marriage may be different. In addition, partnership effects on well-being can differ between different-sex and same-sex couples for two reasons. First, same-sex couples may be less likely to obtain social connections and support for their partnership. Although same-sex marriages were legalized in 2001 in the Netherlands, they may still not be completely accepted by these couples’ family, relatives, neighbors, or even employers and fellow employees (Badgett 1995; Berg and Lien 2002; Carpenter 2007; Clain and Leppel 2001; Elmslie and Tebaldi 2007; Patacchini et al. 2015). As soon as same-sex partners start cohabiting or get married, their sexual orientation is likely to be disclosed to the public, including their employers and coworkers (Plug and Berkhout 2004). Possible discrimination and unfriendly behavior will directly harm their well-being (Hatzenbuehler et al. 2010; Huebner et al. 2004; Mays and Cochran 2001; McCabe et al. 2010; Meyer 2003). Second, pressure from family and society may force sexual minorities to adjust their behavior, which in turn affects their well-being. For instance, they may refuse to openly enter a partnership, be less likely to adopt a child, shy away from prejudiced occupations (Plug et al. 2014), and bear a higher risk of partnership dissolution. According to Statistics Netherlands (n.d.), more than 30 % of female same-sex couples who married in 2005 had divorced by 2015. The corresponding percentages for male same-sex and different-sex couples are 15 % and 18 %, respectively.^3^ Because of the heterogeneity of their partnership formation and stability, same-sex and different-sex couples may differ in the effect of marital partnership on well-being. The issues of the well-being and marital partnership of same-sex couples are largely unexplored in the literature.

Previous studies have investigated differences in well-being effects from marriage and cohabitation but neglected potential heterogeneity of sexual orientation. To the best of our knowledge, we are the first to investigate whether same-sex and different-sex partnerships differ in their effect on subjective well-being. Being the first country to implement the same-sex marriage law, the Netherlands bears the longest duration and relatively mature evolution of same-sex marriages. Thus, its relevant data are considerably appropriate for our specific research topic. Moreover, the Netherlands is a country with a highly tolerant attitude to same-sex, bisexual, and transgender (LGBT) individuals or sexual minorities. For example, in the Eurobarometer 2015, 91 % of the Dutch respondents agreed with the statement that “same-sex marriages should be allowed throughout Europe,” whereas the average across the 28 EU countries was 61 % (European Commission 2015).

We also study whether partnership effects on subjective well-being are age cohort–specific. Older adults are more likely to be unmarried by remaining cohabiting or dating without making a formal commitment (Brown and Shinohara 2013; Brown et al. 2006; Calasanti and Kiecolt 2007; Cooney and Dunne 2001; Sassler 2010) and by increasingly divorcing (Brown and Lin 2012; Kennedy and Ruggles 2014). Later in life, cohabitation operates as a long-term alternative to marriage. Therefore, the positive well-being effect of cohabitation may be comparable with that of marriage for the older cohort (Brown et al. 2012; King and Scott 2005; Vespa 2012; Wright and Brown 2017). However, older adults may also prefer to protect the wealth they have accumulated over their lifetime rather than pool resources with their partner (Brown et al. 2012). Cohabitation allows them to retain financial and economic autonomy (Brown et al. 2016; Chevan 1996; Hatch 1995). Moreover, older adults may be less willing to provide caregiving to a partner at later stages of their life. Cohabitation does not explicitly expect this kind of responsibility as marriage does (Talbott 1998). Therefore, the positive well-being effect of cohabitation could be smaller than that of marriage for older adults. Our study adds to the literature on whether the well-being impact of cohabitation is similar to that of marriage for different age cohorts.

Finally, we analyze whether the well-being effects are symmetric for partnership formation and partnership dissolution. Symmetry implies that partnership formation and partnership dissolution have similar magnitudes but opposite signs. Intuitively, at the beginning of a partnership, a couple is enjoying intimacy and mutual trust (Michael 2004), and thus partnership formation has a positive effect on well-being (Lucas and Clark 2006; Lucas et al. 2003). However, as time goes by, a partnership may be confronted with difficulties and face a breakup. Therefore, partnership dissolution may have a negative effect on the well-being of the individuals involved. Only a handful of studies have examined the well-being gain produced by partnership formation and the well-being loss resulting from a partnership dissolution simultaneously, typically finding strong effects of partnership dissolution (Kalmijn 2017; Simon 2002; Strohschein et al. 2005; Williams and Umberson 2004). However, these studies have not rigorously tested whether partnership formation and dissolution have symmetric effects on well-being. Hence, our study is one of the first to systematically compare every entry-exit pair among different partnership transitions, examining whether the effects within every pair are symmetric.

Our contribution to the literature on partnership and well-being is threefold. First, we establish the causal effects of marriage and cohabitation on subjective well-being. Second, we systematically test the symmetry of partnership formation and dissolution. Third, and most important, we examine well-being effects of same-sex partnerships. We confirm the results from previous studies that the well-being gains of marriage are larger than those of cohabitation. We find that these effects are homogeneous to sexual orientation. We also find gender differences in the well-being effects of same-sex partnerships: females are happier cohabiting, whereas marriage has a stronger well-being effect on males.

## Conceptual Background

### Theoretical Framework

Traditionally two competing models explain the mechanisms through which partnership formation and partnership dissolution affect well-being: the long-term resource accumulation model and the short-term crisis adaptation model.

The long-term resource accumulation model argues that the well-being gains of partnership formation accumulate over time rather than manifest immediately. As a partnership proceeds, the couple invests more resources in terms of shared tangible property (income, real estate, combined families, and mutual friends) and intangible capital (intimacy, trust, commitment, and family responsibilities) (Kamp Dush and Amato 2005; Rhoades et al. 2011; Rusbult 1980). Thus, partnership ties become stronger over time, and the positive well-being effect increases with partnership duration (Kalmijn 2017; Waite and Gallagher 2000). Likewise, well-being losses of partnership disruption will materialize gradually. Additionally, the loss of the gradually accrued investment in the previous partnership makes it difficult for divorcees’ well-being to recover (Stanley et al. 2006). Simpson (1987) showed that after breaking up a longer partnership, people feel higher levels of distress over a longer period than individuals who break up after a short partnership. The resource model has some variants—such as the investment model (Rusbult 1980), role theory (Pearlin 1999), and chronic strain theory (Amato 2000)—all of which share the similar idea of gradual well-being promotion and deterioration in the long run during partnership formation and dissolution, respectively.

The short-term crisis adaption model asserts that the stress around partnership disruption is only temporary, and divorcees are able to recover or adjust quickly. Thus, the initial negative well-being effect will fade with time (Acock and Demo 1994; Booth and Amato 1991; Pearlin 2009; Stroebe et al. 2007). Moreover, Wheaton (1990) claimed that partnership disruption, as a stressful event, actually alleviates the stress of sustaining an unsuitable, low-quality partnership, so the breakup distress is only short-term. Similarly, the positive well-being effect of partnership formation is only temporary. Partnered individuals increase well-being only short term, thereafter adapting back to their original, pre-partnership level of well-being determined by stable internal characteristics, such as personality (Anusic et al. 2014; Lucas and Clark 2006; Lucas et al. 2003; Musick and Bumpass 2012; Soons et al. 2009). Other variants of the crisis model include adaptation theory (Diener et al. 2006; Lucas et al. 2003), the stressful-event-as-stress-relief model (Wheaton 1990), and set-point theory (Anusic et al. 2014).

The theory of the second demographic transition (Lesthaeghe 2007) and the ideational perspective (Lesthaeghe and Surkyn 1988) argue that in countries where citizens’ physiological and safety needs have been met, society shifts to valuing self-actualization and individual autonomy. If partnerships support this kind of self-actualization and individual autonomy, partners in the union will enjoy the well-being gains; otherwise, partners will not have these well-being gains or may even have well-being declines. Similarly, Finkel et al. (2014) argued that in modern society, young people hold increasingly high expectations and standards of marriage, such as personal growth in the marital union. The newly marrieds will feel disappointed if marriage does not meet their high expectations and standards of marriage; hence, their well-being may not change substantially or may even decline after getting married.

### Gender Differences

A few studies have explored gender differences in these well-being effects. Men and women seem to be affected in a similar pattern by marital statuses and transitions (Kalmijn 2017; Strohschein et al. 2005; Williams 2003). However, gender differences in the levels of these effects have been found especially for marital dissolution (Simon 2002; Umberson 1992; Williams and Dunne-Bryant 2006). Simon (2002) and Williams and Dunne-Bryant (2006) found that divorce entails a stronger depression for women than for men and a more significant reduction in psychological well-being for women with young children than for their male counterparts. On the contrary, Kalmijn (2017) and Williams and Umberson (2004) found that marital dissolution undermines life satisfaction and self-reported health more for men than for women. Blekesaune (2008) found that divorce elevates more distress for mothers than for fathers. These differences may be attributed to different social roles of men and women in a partnership (Umberson 1992) or simply to different responses to marital transitions between men and women (Simon 2002).

Gender differences exist between marriage and cohabitation as well, although the results are quite mixed. Wright and Brown (2017) concluded that married and cohabiting men enjoy similar well-being gains compared with dating and single men, but they noted no considerable well-being differences among marital statuses for women. However, Brown et al. (2005) found that among middle-aged and older adults, married women have identical depression scores as cohabiting women and men, but their scores are all higher than those of married men. Mernitz and Kamp Dush (2016) found that among young adults, marriage benefits emotional health for both men and women, whereas cohabitation benefits only women, and these gender differences were detected for first unions only. Kamp Dush (2013) found that among parents who experience union disruption, depressive symptoms of previously married mothers—but not cohabiting mothers—return to predivorce levels; and depressive symptoms of previously married fathers increase more than those of cohabiting fathers. Avellar and Smock (2005) concluded that the dissolution of cohabitation entails a moderate decline for men’s economic situation but hurts women’s economic standing much more intensely.

Cohabitation may have smaller positive effects on well-being than marriage. Because the former is usually regarded as a trial marriage, cohabitants may invest lower levels of tangible and intangible capital (Michael 2004) in their partnership than married couples do (Nock 1995; Soons et al. 2009; Stanley et al. 2004). Cohabitation, as merely a trial marriage, may exert weaker causal protective effects than marriage does in terms of production and consumption complementarities, social connections, and social controls (as discussed earlier). Moreover, cohabitation bears higher disruption rates and lower expectations for future relationship stability than marriage. Therefore, cohabitation dissolution may less intensely affect well-being than marriage dissolution (Blekesaune 2008; Kamp Dush 2013). Recovery from cohabitation disruption may be also faster than recovery from divorce.

### Sexual Minorities

The literature on the well-being effects of different types of partnerships for sexual minorities is limited, and a distinction based on gender is even more rare. According to the minority stress theory (Meyer 2003), sexual minorities in a relationship experience stress when interacting with other people, so they respond with coping strategies, including concealing their relationship (Rostosky et al. 2007). The minority stress may shrink the well-being gap between marriage and cohabitation. Nonetheless, it is also possible that only sexual minorities with lower levels of such minority stress select themselves into marriage. This selection will enlarge the well-being gap between marriage and cohabitation.

Empirically, Fingerhut and Maisel (2010) claimed that having a domestic partnership (closer to marriage than to cohabitation legally) alleviates negative effects of stress on life satisfaction for sexual minorities. Riggle et al. (2010) found that sexual minorities in legally recognized relationships report less psychological distress and higher well-being than those in committed relationships and that a similar gap exists between those in committed relationships and singles. Wight et al. (2013) found that sexual minorities in marriage and domestic partnerships have identical levels of psychological distress, which are lower than those of sexual minority singles and higher than those of different-sex married couples. Gorman et al. (2015) discovered that only among different-sex couples do women report significantly different physical health from men; among sexual minorities, gender differences in physical health do not exist.

## Methodology Review

The methodology to establish a relationship between partnership and well-being has evolved as researchers have made efforts to conquer more challenging questions: going from association to causality and accounting for reverse causality. Three types of studies can be distinguished with increasing degrees of complexity of the analysis. The first type uses cross-sectional data, focusing on correlation between partnership and well-being. Gove and Shin (1989), White (1992), Mastekaasa (1995), and Diener and Suh (1997) conducted such an analysis for the United States, Canada, Norway, and multiple countries together, respectively. They confirmed the positive association between subjective well-being and marriage across countries and cultures. Kurdek (1991) and Mastekaasa (1995) showed that cohabitation is also positively correlated with subjective well-being in some countries. None of the studies in this category addressed the issue of causality: that is, they did not distinguish selectivity from causality or consider possible reverse causality.

The second type tries to remove the selection effect whereby happier people are more likely to enter a partnership. The selection effect is due to individual-specific unobserved heterogeneity, such as personality: extroverted people may be both happier and more likely to find a partner. Static fixed*-*effects studies take into consideration individual time-invariant unobserved heterogeneity (e.g., Averett et al. 2013; Ferreri Carbonell and Frijters 2004; Musick and Bumpass 2012; Stutzer and Frey 2006). Most of these studies found that partnership, including marriage and cohabitation, increases the well-being of individuals who enter a partnership. However, this conclusion is not universal. For instance, Averett et al. (2013) showed that marriage leads to a higher body mass index, to overweight and obesity, and to less exercise. These studies found evidence of a positive selection effect. Chapman and Guven (2016) employed data from the United States, the United Kingdom, and Germany and introduced the quality of marriage as an additional explanatory variable. They discovered that the positive effect of marriage on happiness is driven by happy marriages. For couples who are not happily married, marriage has a negative effect on happiness.

The third type of studies focuses on addressing potential reverse causality—that is, a shock to the well-being of an individual leads to a jump of the likelihood of entering a partnership for that individual. Lillard and Panis (1996) employed a simultaneous-equation framework using proportional hazards for health and marital separations. The correlation of the errors of the two equations captures the selection effect. They attempted to address reverse causality by introducing instrumental variables in the health equation. Using a similar measure, van den Berg and Gupta (2015) found that men generally enjoy a protection effect of marriage, whereas women benefit from marriage only after childbearing age. Ali and Ajilore (2011) applied propensity score matching to obtain a counterfactual outcome and correct for selection on observable characteristics. Their results showed that marriage indeed reduces risky health behaviors and thus improves well-being. Two studies by Kohn and Averett (2014a, 2014b) assumed sequential reverse causality from current well-being to the partnership choice in the next period. Their first study used a dynamic fixed-effects model with internal instruments advocated by Blundell and Bond (1998) to account for reverse causality. Their second study exploited a random-coefficient mixed logit model to estimate the unobserved heterogeneity associated with both health and relationship choice, enabling them to disentangle the reverse causality due to this unobserved heterogeneity. Both studies found that marriage and cohabitation benefit health similarly.

## Data and Statistical Model

### Data

Our research is based on data from the Longitudinal Internet Studies for the Social Sciences (LISS) panel administered by CentERdata (www.lissdata.nl). The panel is a random sample of households drawn from the Dutch population consisting of more than 6,500 households, more than 10,000 individuals, and 93 monthly waves over the period November 2007–July 2015.

With information of partnered household heads and their marital or cohabiting partner, we identify the sexual orientation of each individual by comparing one’s gender with that of his or her partner (see the online appendix, section A, for details). Thus, individuals who were always single during the period of observation are not included in our analysis that includes sexual orientation.^4^ First, we investigate the effect of any partnership on subjective well-being. Then, we study whether marriage has a different effect on subjective well-being than cohabitation. As society has become more and more tolerant and people have become increasingly open-minded about partnerships, cohabitation has become considerably popular and a soaring tendency in partnerships, especially in the Netherlands (Latten and Mulder 2013). Given the rapid expansion of cohabitation and its distinction from other marital statuses, it is reasonable to isolate it as a different category.

Our sample contains 27,779 observations, 425 of which are individuals who entered a same-sex relationship.^5^ The sample size of sexual minorities is comparatively small, but it matches the estimated share of sexual minorities in the population (Bakker et al. 2009; Sandfort et al. 2006). In comparison with other studies, our sample of sexual minorities is quite large.

**Fig. 1:**
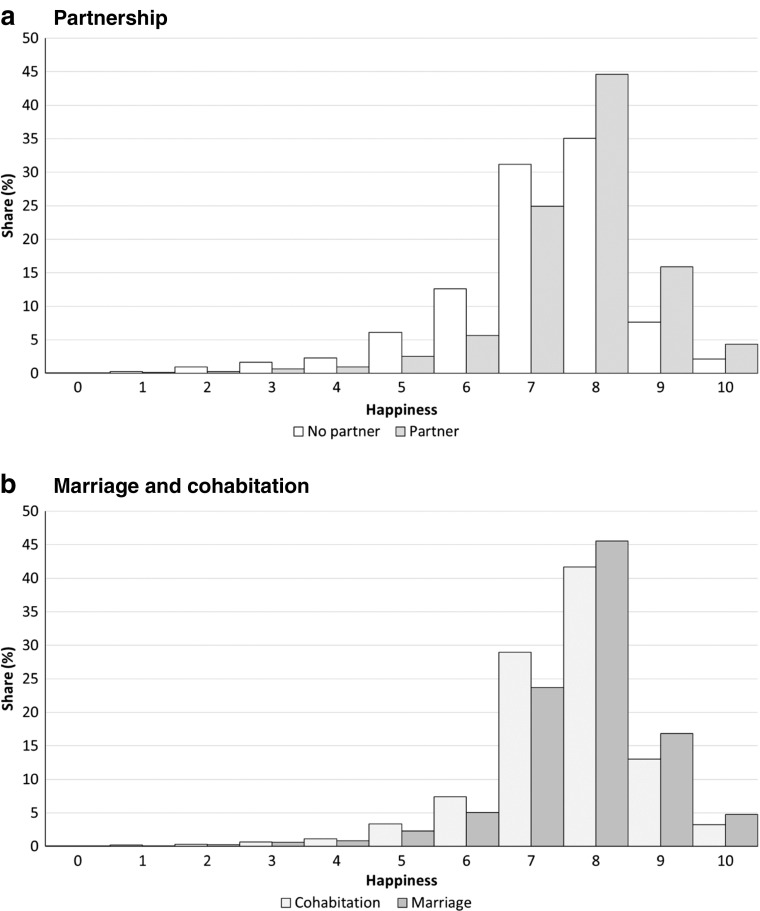
Well-being and partnership

Our indicator of well-being is based on the question, “On the whole, how happy would you say you are?” Responses are provided on an ordinal scale from 0 (totally unhappy) to 10 (totally happy). Panel a of Fig. 1 illustrates the well-being distribution by partnership status. Few respondents reported happiness below 5 on the 0–10 scale. A higher percentage of individuals in the relatively lower responses of 5, 6, and 7 were nonpartnered, whereas a higher percentage of individuals in the higher responses of 8, 9, and 10 were partnered. Apparently, couples are happier than nonpartnered individuals. Panel b of Fig. 1 further distinguishes marriage from cohabitation in the partnership forms. Compared with married couples, cohabitants account for higher proportions in the happiness response score groups of 5, 6, and 7 but lower proportions in the responses of 8, 9, and 10. Generally speaking, partners are happier if they are married compared with cohabiting. Nonetheless, the differences between various types of individuals in Fig. 1 are all unconditional and can only be suggestive of a causal effect of partnership on evaluative happiness.

Table 1 gives an overview of average well-being distinguished by marital status and sexual orientation. The last column in the table confirms the findings in Fig. 1. On the 0–10 scale, nonpartnered individuals have an average score of 7.12, and partnered individuals have an average score of 7.71. On average, married couples score 7.76, and cohabitants score 7.56. Comparing the first two columns of Table 1, irrespective of the marital status, sexual minority individuals are happier on average, although the difference is substantial only in the period when they are single. Because the number of observations of singles is rather small, we make no distinction among never married, separated, divorced, and widowed.^6^

**Table 1:** Subjective well-being by marital status and sexual orientation: Averages, with number of observations in parentheses

	Different Sex		Same-Sex		Unknown		Average	
a. Partnership								
No partner	6.98	(801)	7.65	(34)	7.14	(5,224)	7.12	(6,059)
Partner	7.73	(19,104)	7.76	(391)	7.55	(2,225)	7.71	(21,720)
b. Marriage and Cohabitation								
Marriage	7.76	(16,043)	7.83	(220)	7.81	(369)	7.76	(16,632)
Cohabitation	7.58	(3,061)	7.68	(171)	7.50	(1,856)	7.56	(5,088)

*Notes:* The category “Unknown” exists because either (1) these individuals have always been single, or (2) if they have ever been partnered, their partners did not participate in the survey, and thus their sexual orientation cannot be identified. See online appendix, section A for details.

The partnership transitions are displayed in Table 2, which shows a persistent stability in partnership status. Among the 6,702 individuals in our sample, only 614 partnership transitions happened over a period of five years. Transitions from cohabitation account for the largest fraction, at more than twice the transition rates from each of the other two marital statuses. Most of the cohabitants broke up rather than entered a marriage. More than twice the number of single individuals switched to cohabitation than to marriage. Given the number of observations of these marital statuses in the sample, marriage is considerably more stable compared with cohabitation.

**Table 2:** Number of partnership transitions

	Married	Cohabiting	Single	Total
Married	––	72	61	133
Cohabiting	159	––	180	339
Single	44	98	––	142
Total	203	170	241	614

*Note:* Based on 27,779 observations of 6,702 individuals over five years.

### Statistical Model

Subjective well-being is measured on an ordinal scale from 0 to 10. To account for time-invariant unobserved personal characteristics, we use a linear fixed-effects model even though the dependent variable in such a model is supposed to be cardinal. As Ferreri Carbonell and Frijters (2004) and Stutzer and Frey (2006) indicated, when analyzing happiness and life satisfaction, the linear fixed-effects model performs as well as the fixed-effects ordered logit model.^7^ Our model is specified as follows:

1 $$ {h}_{it}={p}_{it}^{\hbox{'}}{\upbeta}_p+{x}_{it}^{\hbox{'}}{\upbeta}_x+{\upalpha}_i+{\upvarepsilon}_{it}, $$

where *i* (*i* = 1*,* 2, . . . , *n*) refers to individuals; *t* (*t* = 1*,* 2, . . . , *T*) represents *years*; *p* is either the *partnership* dummy variable or a dummy vector of different marital statuses, including *married* and *cohabiting*, with *single* as the reference; *h* denotes *well*-*being* measured on a scale from 0 to 10; and **x** represents the vector of covariates that may be correlated to both *partnership* and *well*-*being*, such as *drinking* and *smoking behavior* (Clark and Etilé 2006), *body mass index* (Clark and Etilé 2011), and *physical problems* (Graham et al. 2011; Kohn and Averett 2014b), as well as demographic and socioeconomic variables, such as *the number of children living at home*, whether the respondent is a *homeowner*, *log of personal net monthly income* (in euros), whether the respondent holds a *college degree*, and *age cohort dummy variables*. Finally, α_*i*_ represents individual-specific time-invariant effects. The error terms ε_*it*_ are assumed to have a mean of 0 and to be independent of ${p}_i^{\hbox{'}}=\left({p}_{i1}^{\hbox{'}},\dots, {p}_{iT}^{\hbox{'}}\right)$ and ${x}_i^{\hbox{'}}=\left({x}_{i1}^{\hbox{'}},\dots, {x}_{iT}^{\hbox{'}}\right)$. We remove time-invariant unobserved heterogeneity that may affect both partnership and well*-*being, such as *personality*, by subtracting individual sample means.

We start our analysis with a pooled cross-section analysis, ignoring individual fixed effects. Conditional on observed characteristics, we estimate the association between partnership and well-being. The association combines the effect of selectivity and the causal effect of partnership on well-being. Then, we introduce individual fixed effects to remove the effect of selectivity, thus establishing a causal effect. In a separate analysis, we also investigate the presence of reverse causality by relating current well-being to future partnership.

## Parameter Estimates Subjective Well-being

### Baseline Estimates

The relevant parameter estimates of our fixed-effects model are displayed in Table 3. The two columns show the partnership effect on happiness for males and females separately. To indicate the importance of considering individual fixed effects, we present ordinary least squares (OLS) parameter estimates in panel a.^8^ There, partnership elevates subjective well-being by 0.60 for men and 0.45 for women, approximately 0.5 points on an 11-point scale. With the fixed-effects setting in panel b, partnership also has a positive effect on happiness, and the difference between males and females is small. Comparing estimates of panels a and b reveals that the OLS estimates are partly driven by positive selection, such that happier individuals are more likely to have a partner. Nevertheless, after we remove this selection effect with the fixed-effects model, there is still a significant increase in well-being related to partnership of approximately 0.25. So, the effect of partnership on subjective well-being and the selection effect explain approximately 50 % of the positive association between partnership and well-being, respectively. Although well-being is measured on a scale from 0–10, few respondents report a well-being response lower than 6, and few individuals report a 10. In relative terms, an increase of 0.25 over a range of 6–9 is quite substantial.

**Table 3:** Parameter estimates for the effects of partnership on subjective well-being: OLS and individual fixed effects

	Males		Females	
OLS				
a. Partner	0.60**	(0.06)	0.45**	(0.05)
Individual Fixed Effects				
b. Partner	0.26**	(0.07)	0.27**	(0.07)
c. Partnership by sexual orientation				
Different-sex partner (β_*dsp*_)	0.27**	(0.08)	0.27**	(0.08)
Same-sex partner (β_*ssp*_)	0.25	(0.31)	0.71^†^	(0.42)
*p* value (β_*dsp*_ = β_*ssp*_)	0.940		0.303	
d. Marriage versus cohabitation				
Marriage (β_*m*_)	0.33**	(0.08)	0.39**	(0.08)
Cohabitation (β_*c*_)	0.21**	(0.07)	0.21**	(0.07)
*p* value (β_*m*_ = β_*c*_)	.086^†^		.004**	
e. Marriage versus cohabitation by sexual orientation				
Different-sex marriage (β_*dsm*_)	0.32**	(0.09)	0.44**	(0.09)
Different-sex cohabitation (β_*dsc*_)	0.25**	(0.08)	0.17*	(0.08)
*p* value (β_*dsm*_ = β_*dsc*_)	.351		.000**	
Same-sex marriage (β_*ssm*_)	0.69^†^	(0.41)	0.15	(0.51)
Same-sex cohabitation (β_*ssc*_)	0.18	(0.32)	0.85^†^	(0.42)
*p* value (β_*sm*_ = β_*sc*_)	.094^†^		.058^†^	

*Note:* Panels a, b, and d contain 27,779 observations of 3,088 males and 3,617 females; panels c and e contain 20,330 observations of 2,275 males and 2,526 females. Standard errors are shown in parentheses.

^†^*p <* .10; *p < .05; **p < .01

In panel c of Table 3, we explore whether partnership effects are different for same-sex and different-sex couples. For males, the effect of having a same-sex partner is approximately the same as that of having a different-sex partner. For females, the well-being effect of having a same-sex partner is much higher than that of having a different-sex partner. However, like in the case of males, we cannot reject that partnership exerts identical influences on happiness for females in same-sex and different-sex couples.

Panel d of Table 3 shows that marriage makes couples happier than does cohabitation.^9^ We compare the effects of marriage and cohabitation to that of being single. Later herein, we systematically analyze the dynamics or transitions among different partnership statuses. The positive effect of marriage on well-being is stronger for women than for men. The well-being effect of cohabitation is the same for both genders.

In panel e of Table 3, we distinguish different-sex and same-sex marriage and cohabitation. For different-sex partnerships, the effects of marriage and cohabitation are similar to those presented in panel d. For same-sex male partnerships, the well-being effects of marriage are substantially larger than those of cohabitation. Results for same-sex female partnerships are the opposite: that is, the well-being effects of cohabitation are substantially larger than those of marriage.

All in all, we conclude that partnership has a positive effect on subjective well-being and that this positive effect is statistically identical for same-sex and different-sex couples. Given the significant effect of marital partnership during the short survey period of five years, our results support the idea that the well-being benefits manifest in the short term, as the crisis model (Booth and Amato 1991; Pearlin 2009) and adaptation theory (Diener et al. 2006; Lucas et al. 2003) would suggest.

### Reverse Causality

In the analysis of the effects of partnership dynamics on well-being, there is a possibility of selectivity or reverse causality, or both. With the linear fixed-effects model, we remove selectivity due to individual-specific unobserved heterogeneity related to both partnership and happiness. However, the linear fixed-effects model does not account for possible reverse causality—that is, the possibility that an individual whose happiness increases is more likely to find a partner. A person who becomes happier and more satisfied with his or her life may appear more confident and be more willing to socialize, making the person more attractive and approachable in the partnership market. Similarly, a person who experiences depression may have difficulty finding a partner (Sandberg-Thoma and Kamp Dush 2014).

To investigate whether reverse causality is an issue, we study whether single people are more likely to be partnered later on, as their happiness changes over time because of some shock. We estimate a fixed-effects model in which the dependent variable is whether an individual is *partnered*, and the independent variables are *happiness* in an earlier period and the same covariates as before. If reverse causality existed, we would expect that a higher level of happiness makes partnership formation later on more likely. We use different lags for happiness to allow for effects that materialize quickly or more slowly.

Table 4 displays the relevant parameter estimates of lagged happiness. Row a shows that a positive shock to happiness of an individual who was single does not improve his or her probability to enter a partnership one year later. Rows b–d present that also after two, three, or four years, there is no effect. None of the results are sizable or significant except the coefficient in row c for women. Although significant at 5 % significance level, the magnitude of 1 % is still negligible. From this, we conclude that reverse causality from happiness to future partnership dynamics is not an issue.

**Table 4:** Parameter estimates of the effects of subjective well-being on partnership: Individual fixed effects

	Partnered_*t*_			
	Males		Females	
a. Happiness_*t −* 1_	–0.002	(0.005)	–0.000	(0.003)
b. Happiness_*t −* 2_	–0.003	(0.006)	0.002	(0.004)
c. Happiness_*t −* 3_	0.004	(0.007)	–0.010*	(0.004)
d. Happiness_*t −* 4_	0.004	(0.009)	–0.007	(0.006)

*Note:* Standard errors are shown in parentheses. Covariates and a constant are included in every model but are not shown for parsimony.

*p < .05

### Symmetry

Partnership formation and partnership disruption may have different effects on subjective well-being both in sign and magnitude. Therefore, it is interesting to distinguish between entering a partnership and ending it and then to test whether their effects are symmetric. We introduce a *single to partnered* dummy variable with value of 1 in case of partnership formation and 0 otherwise. Likewise, the *partnered to single* dummy variable is 1 in case of partnership dissolution and 0 otherwise.

Panel a of Table 5 presents seemingly asymmetric effects during partnership formation and during partnership dissolution. The first term, *single to partnered*, refers to the effect when a partnership forms; the second term, *partnered to single*, refers to the effect when a partnership dissolves. In both columns, partnership formation and disruption have opposite effects on the subjective well-being for both men and women. For example, males who transition from singlehood to partnership experience an average increase in well-being of 0.18. If they break up and become single, they face a decrease in well-being of 0.30. To formally check whether the effects are identical in magnitude during partnership formation and disruption, we conduct the pair symmetry test with the null hypothesis such that the absolute values of the coefficients of the two transition variables are equal. The *p* value of the test indicates that we cannot reject that the effects are symmetric.

**Table 5:** Parameter estimates of the effects of partnership on subjective well-being: Asymmetry of partnership formation and dissolution

	Males		Females	
a. Partnership				
Single to partnered (β_*sp*_)	0.18^†^	(0.09)	0.17	(0.10)
Partnered to single (β_*ps*_)	–0.30**	(0.09)	–0.29**	(0.08)
*p* value (β_*ps*_ = *−*β_*sp*_)	.339		.351	
b. Marriage and Cohabitation Transitions				
Single to married (β_*sm*_)	0.17	(0.16)	0.28	(0.20)
Married to single (β_*ms*_)	0.25	(0.15)	–0.00	(0.13)
*p* value (β_*sm*_ = β_*ms*_)	.722		.249	
Single to cohabiting (β_*sc*_)	0.06	(0.11)	0.05	(0.12)
Cohabiting to single (β_*cs*_)	–0.18^†^	(0.10)	–0.14	(0.09)
*p* value (β_*cs*_ = *−*β_*cs*_)	.418		.561	
Cohabiting to married (β_*cm*_)	0.06	(0.10)	0.08	(0.09)
Married to cohabiting (β_*mc*_)	–0.31*	(0.15)	–0.02	(0.11)
*p* value (β_*cm*_ = *−*β_*mc*_)	.152		.660	
*p* value (β_*sm*_ *−* β_*ms*_ = β_*sc*_ + β_*cs*_ = β_*cm*_ + β_*mc*_ = 0)	.429		.599	

*Notes:* Column 1 contains 12,955 observations of 3,088 men; column 2 contains 14,824 observations of 3,617 women. Standard errors are shown in parentheses.

^†^*p <* .10; *p < .05; **p < .01

Partnership is heterogeneous in the sense that it includes informal cohabitation and formal marriage. The subjective well-being derived from cohabitation and marriage is likely to be different. Thus, we further investigate the symmetries of transitions among marriage, cohabitation, and singlehood. Panel b of Table 5 displays the effects on subjective well-being of several types of partnership dynamics. For example, entering marriage does not seem to raise subjective well-being for cohabiting couples, whereas going from marriage to cohabitation significantly reduces men’s happiness but does not affect women’s happiness.^10^ Marriage provides a tighter, more socially recognized and enforceable contract than cohabitation, and this seems to be the case especially for males. Nevertheless, for these more elaborate dynamics among singlehood, cohabitation, and marriage, although the symmetries still hold, most of the estimates are insignificant. This may be due to the small number of observations in each transition (see Table 2). The estimation of the partnership dynamics also provides evidence for the short-term crisis model or adjustment theory. During partnership formation, subjective well-being improves quickly; during partnership dissolution, subjective well-being is harmed immediately.

### Age Cohort Differences

Marital partnership may have different meanings for younger and older individuals. For instance, younger adults usually view cohabitation as a trial marriage, whereas older individuals may think of cohabitation as a long-term substitute for marriage (Brown et al. 2012; King and Scott 2005; Vespa 2012; Wright and Brown 2017).

To investigate potential heterogeneity in the effects of partnership on well-being, we test for differences by age. Kohn and Averett (2014b) distinguished individuals under 45 and over 45 and indeed found different relationship effects for the two subsamples. Following their idea, we divide the sample into two age cohorts: people born before 1962 (46 years old in the first wave of the survey in 2008) and after 1962. The relevant parameter estimates are displayed in Table 6. Panel a shows that partnership increases happiness for men born before 1962 but not for women in the same age cohort. Both men and women in the older cohort obtain larger well-being gains from marriage than from cohabitation. Panel b displays that partnership exerts a positive influence in the younger cohort, and this is true for both marriage and cohabitation. For the younger cohort, the happiness benefits from marriage are larger than those from cohabitation, but the difference is not statistically significant.

**Table 6:** Parameter estimates of the effects of partnership on subjective well-being by age cohort

	Males		Females	
a. Born Before 1962				
Partner	0.28*	(0.12)	0.17	(0.15)
Marriage vs. cohabitation				
Marriage (β_*m*_)	0.36**	(0.12)	0.31*	(0.16)
Cohabitation (β_*c*_)	0.13	(0.14)	–0.10	(0.17)
*p* value (β_*m*_ = β_*c*_)	.044*		.000**	
b. Born in 1962 or Later				
Partner	0.25**	(0.09)	0.30**	(0.08)
Marriage vs. cohabitation				
Marriage	0.30**	(0.11)	0.37**	(0.10)
Cohabitation	0.23**	(0.09)	0.28**	(0.08)
*p* value (β_*m*_ = β_*c*_)	.515		.313	

*Notes:* Panel a contains 15,395 observations with 1,704 men and 1,773 women; panel b contains 12,384 observations with 1,385 men and 1,845 women. Standard errors are shown in parentheses.

*p < .05; **p < .01

These findings raise an interesting question. Why does cohabitation benefit the younger age cohort but not the older one? We speculate that older adults may prefer to protect the wealth they have accumulated over their lifetime rather than pool the resources with their partner (Brown et al. 2012), and cohabitation allows them to retain financial and economic autonomy that would not be possible in marriage (Brown et al. 2016; Chevan 1996; Hatch 1995). Furthermore, older adults, especially older women, may be less willing to provide caregiving to a partner at a later stage of their life, and cohabitation does not explicitly encourage this kind of responsibility as marriage does (Talbott 1998). Another possible explanation is that for people born before 1962, cohabitation was still not widely accepted when they entered the partnership market. The social attitude regarding cohabitation may also have influenced their individual attitudes. Although they later chose to cohabit, they still did not regard cohabitation as similar to marriage. On the contrary, when individuals in the younger age cohort entered a partnership, societal attitudes regarding cohabitation were quite tolerant. Currently, cohabitation is a more popular partnership choice than marriage.

## Conclusions

Many studies have found positive well-being effects of a partnership, for which there are various explanations. Partnered individuals may gain from production complementarities, division of labor or consumption, and investment complementarities. Couples may also benefit from economies of scale by pooling resources, jointly consuming public goods and investing in children, and sharing leisure activities. A partnership may strengthen and expand social relationships. Finally, a partnership may introduce social control and mutual supervision.

We analyze Dutch panel data to investigate whether partnership has a causal effect on subjective well-being, finding that this is indeed the case. We do not find evidence for reverse causality, which occurs if a positive shock to one’s well-being induces partnership formation. As in a few previous studies, we find that well-being gains of marriage are larger than those of cohabitation, a result that may be related to different investment levels of tangible and intangible capital. We also find that the well-being effects of partnership formation and disruption are symmetric. Because our panel covers a five-year period, this finding supports the prediction based on crisis model and adaptation theory that the well-being effects of marital partnership transitions manifest in the short term rather than that they need a long time to accumulate. Furthermore, we find that marriage improves well-being for both younger and older cohorts, whereas cohabitation benefits only the younger cohort. This finding may be due to the weaker desire of pooling economic resources and lower willingness of caregiving for older cohabitants. Alternatively, it could be the result of different social acceptance of cohabitation when older individuals initially entered the partnership market a long time ago; although they later on chose to cohabit, perhaps older individuals still do not regard cohabitation as similar to marriage.

We contribute to the literature by studying partnership dynamics, investigating reverse causality and establishing cohort-specific differences in well-being effects, but our main contribution is illuminating well-being effects of same-sex partnerships. We find that these effects are similar to those of different-sex partnerships. This may seem surprising because of possible discrimination against sexual minorities after their sexual orientation is disclosed. Perhaps thanks to the effective implementation of education and policy on marriage equality and respect for sexual minorities, this prejudice against sexual minorities does not prevail in the Netherlands. Although same-sex and different-sex partnerships overall have similar effects on well-being, we do find gender differences in the well-being effects of same-sex partnerships. Females are happier cohabiting, whereas marriage has a stronger well-being effect on males. We can only speculate about the reasons for this difference given that the literature on the well-being effects of different types of partnerships for sexual minorities is limited. Perhaps marriage provides a tighter, more socially recognized and enforceable contract than cohabitation, especially for male same-sex partnerships. For female same-sex partnerships, this seems to be less of an issue.

We are confronted with a few difficulties in the current study. First, the analysis is based on a short panel, so we are unable to examine whether the well-being effects of partnership dynamics will persist in the long term. The crisis model argues that the effects are temporary, whereas the resource model suggests that the effects need a long time to materialize. To investigate which of the models is more realistic, a longer panel would be more helpful. Second, to analyze heterogeneity of sexual orientation in well-being effects of partnership dynamics in more detail, a larger data set is needed. The number of partnership transitions and the size of same-sex sample are still relatively small in our data. Because of these limitations, our parameter estimates for same-sex partnerships are imprecise. Third, although we include a number of time-varying covariates and apply the fixed-effects model to account for time-invariant unobservable characteristics, we cannot completely resolve the concern of the possible time-varying confounding unobservable factors. If panel data contain information on the nature and magnitude of exogenous shocks to partnership market, we would be able to exploit such a shock to draw a more compelling causal conclusion.

## Electronic supplementary material

ESM 1 (PDF 204 kb)

## Footnotes

1 The literature regards subjective well-being as a substitute for happiness (Diener et al. 2009). We use the two terms interchangeably.

2 There could be adverse selection, too, if individuals with inferior well-being are more likely to seek actively for partnership because of the protection it offers.

3 The differences in divorce risks between same-sex partnerships and different-sex partnerships may be attributed to different factors. Same-sex couples are less likely to have children in their household, and children and the investment in children usually help stabilize a partnership (Andersson et al. 2006). Another risk factor of divorce for same-sex couples is a big age difference (Noack et al. 2005). As in the Netherlands, same-sex marriages of women in Norway and Sweden have the highest divorce risks (Andersson et al. 2006; Noack et al. 2005). The reasons for gender differences in divorce risks of same-sex partnerships are not clearly addressed in the literature. One explanation may be different levels of minority stress (Meyer 2003). Moreover, the so-called open relationship or open marriage may prevail more in same-sex couples of men and less so in same-sex couples of women compared with different-sex couples.

4 If the reason for remaining single is not intentional, this does not bias our results. However, if singles did not enter a partnership because they would not benefit in terms of well-being, we will overestimate the well-being effects of partnership formation. Nevertheless, it is also possible that these singles have lower well-being levels than people who experienced at least one partnership during the sample period and could have benefited more than average from partnership formation. If that is the case, the well-being effects of partnership will be underestimated in our study.

5 The definitions and descriptive statistics of the relevant variables in the main models are provided in Tables S1 and S2 in online appendix, section A2.

6 As shown in Table 1, our sample includes 34 observations of the singlehood period for sexual minorities. Among them, 9 observations are for the divorced phase, and 25 observations are for the never-married period. Of the 801 observations of the singlehood period for the sexual majority, 21 observations are for the phase of separation from a cohabitation, 330 observations are for the period of divorce from marriage, 49 are for a widow phase, and 401 are for the period of being never-married. In addition, we cannot establish sexual orientation of 5,224 observations of singles. The latter group of singles is used only in the first part of our analysis.

7 This is also the case in our analysis. By way of sensitivity analysis, we estimated a fixed-effects ordered logit model and find very similar results.

8 We also run the OLS models on the subset of people who changed partnership status during the survey period as a robustness check. The results are similar to those in panel a of Table 3.

9 We consider panel d in Table 3 as our baseline estimates. Section B in the online appendix presents the parameter estimates of the full baseline model.

10 Interpreting these parameter estimates should be cautious because data limitations require that we ignore partnership transitions within a year.
