# Supplementary material for: Subjective Well-being and Partnership Dynamics: Are Same-Sex Relationships Different?
Source: Demography. 2018 Nov 1;55(6):2299–320. doi: 10.1007/s13524-018-0725-0 (PMC6290656; doi:10.1007/s13524-018-0725-0)
Supplement: Supplementary file 1 — (PDF 204 kb) [file 13524_2018_725_MOESM1_ESM.pdf]

A: Details on our data

A1: Sexual orientation

It is hard to measure an individual's sexual orientation in surveys. There are basically three ways to do this and each method has its limitations. The first method is simply asking for sexual preferences: "Regarding your sexual preference, are you attracted to men or to women?" Answers could be in five categories: one only to men; two especially to men, but to some extent also to women; three as much to men as to women; four especially to women, but to some extent also to men; five only to women. This measure was employed by Plug and Berkhout (2004), and Buser et al. (2015). The second measure of sexual orientation is through sexual activity. Badgett (1995) and Black et al. (2003) used answers to the question "How many males and females did you have sex with?" The third measure of sexual orientation is based on the gender of respondents' partner. This measure was used by Klawitter and Flatt (1998) and Allegretto and Arthur (2001).

The three measures of sexual orientation have their own advantages and shortcomings: sexual preference and past sexual activity ask directly about sexual orientation so they can identify sexual orientation with just cross sectional data even for respondents who are single at the time of the survey. However, they may result in plenty of non-responses because of privacy. Besides, past sexual activity will probably wrongly classify, for example, individuals who participated in different-sex activities a few times but then figured out they prefer same-sex relationships. Data of the gender of respondents' partner are more widely accessible than sexual preference and past sexual activity. Moreover, sexual orientation based on partner's gender is more observable to the respondents' family and employers. Thus, if the researchers want to investigate outside influence related to sexual orientation, this measure is more appropriate. Nevertheless, for respondents who were partnered in none of the waves of the panel, this measure can not detect their sexual orientation. This may lead to sample selection (Plug and Berkhout 2004). The three measures capture different respects of sexual orientation hence are not necessary to be completely consistent. Which measure to use empirically depends on the specific problem to be investigated. We study the effect of partnership on subjective well-being where in part of our analysis we distinguish between different-sex and same-sex relationships. Since such an effect is directly related to the respondents' partner during the partnership, the measure of sexual orientation based on partner's gender is most suitable.

From the background variables in the LISS-panel, we know the position within the household of each of the respondents, i.e., whether they are household head, wedded

partner, cohabiting partner, parent (in law), child living at home, house mate, and family member or boarder. We also know marital status which includes never married, married, separated, divorced, and widowed. Information on the domestic situation includes single without child(ren), single with child(ren), (un)married cohabitation without child(ren), (un)married cohabitation with child(ren), and other. With these variables we are able to identify the sexual orientation of every household head and their partner.

First, we combine the originally 93 monthly waves to construct an initial panel. Second, in the initial panel we keep only the partnered household heads and their (un)wedded partner using the categories of (un)married cohabitation with(out) child(ren) in "domestic situation". Third, we identify the sexual orientation of every partnered individual by comparing one's gender with that of one's (un)wedded partner and record the corresponding person numbers in the same-sex group and different-sex group respectively.[1]

A2: Definitions and descriptives of variables

The subjective well-being indicator is collected annually, while other variables including the partnership dynamics are available on a monthly basis. In our analysis all variables are specified on an annual basis. This means some loss of information, for example, multiple changes in partnership status within a year are ignored. Table 7 provides an overview of the definition of the variables we use in our analysis. Table 8 presents the descriptives of these variables.

B: Parameter Estimates Baseline Model

Table 9 presents a full set of parameter estimates related to Table 3 panel d. The first two rows indicate the effects of marriage and cohabitation, identical to the ones presented in Table 3 panel d. Teenagers (the reference of the age group dummies) appear to have the highest level of happiness. The happiness of men aged 20 to 29 is somewhat lower while from age 30 onward well-being drops even further. However, for females the age gradient is hardly present. The number of children has a negative effect on happiness although only for females this effect is significantly different from zero. Net income has a positive effect on happiness for males but not for females. Physical problems have a

[1]There are two exceptions, bisexuals and trans-genders, which consist of 30 individuals together. Following previous studies (Plug et al. 2014; Buser et al. 2015) we categorize them into same-sex group since they all belong to sexual minorities. In the interpretation and discussion we will use the expressions of same-sex and sexual minorities interchangeably.

negative happiness effect for males and smoking has a positive effect for males. Most of the other variables have no significant effect on happiness.

References

Allegretto, S. A. and Arthur, M. M. (2001). An empirical analysis of homosexual/heterosexual male earnings differentials: unmarried and unequal? *Industrial and Labor Relations Review*, 54(3):631–646.

Badgett, M. L. (1995). The wage effects of sexual orientation discrimination. *Industrial and Labor Relations Review*, 48(4):726–739.

Black, D. A., Makar, H. R., Sanders, S. G., and Taylor, L. J. (2003). The earnings effects of sexual orientation. *Industrial and Labor Relations Review*, 56(3):449–469.

Buser, T., Geijtenbeek, L., and Plug, E. (2015). Do gays shy away from competition? Do lesbians compete too much? *IZA Discussion Paper*, (9382).

Klawitter, M. M. and Flatt, V. (1998). The effects of state and local antidiscrimination policies on earnings for gays and lesbians. *Journal of Policy Analysis and Management*, 17(4):658–686.

Plug, E. and Berkhout, P. (2004). Effects of sexual preferences on earnings in the Netherlands. *Journal of Population Economics*, 17(1):117–131.

Plug, E., Webbink, D., and Martin, N. (2014). Sexual orientation, prejudice, and segregation. *Journal of Labor Economics*, 32(1):123–159.

Table S1: Definitions of Variables

Variable	Definition
Subjective well-being	"On the whole how happy would you say you are?" (score 0-10)
Partnered	Dummy variable if partnered
Married	Dummy variable if married
Cohabiting	Dummy variable if cohabiting
Single	Dummy variable if never married, separated, divorced or widowed
Same-sex	Dummy variable if classified into same-sex group
Children number	Number of living-at-home children
Home owner	Dummy variable if home owner
Net income	Personal net monthly income in Euros
Missing info net income	Dummy variable if net income is missing
College	Dummy variable if with college diploma
Drinking	Dummy variable if drink alcohol during the last seven days
Drinking days	Number of days in the past seven days drink alcohol
Smoking	Dummy variable if smoke now
BMI	Body Mass Index
Physical problem	Number of physical problems diagnosed by physicians
Missing info physical problem	Dummy variable if physical problem is missing
Age20–70p	Age cohort dummies, reference cohort is teenagers

Table S2: Descriptives

	Men			Women		
Variable	Mean	Minimum	Maximum	Mean	Minimum	Maximum
Subjective well-being	7.6	0	10	7.6	0	10
Number of children	0.8	0	7	0.9	0	7
Net income/10^4	0.2	0	16.3	0.1	0	28.6
Drinking days	2.8	0	7	1.9	0	7
BMI	25.7	13.9	64.4	25.4	12.4	81.4
Physical problem	0.8	0	10	0.8	0	18
Percentages						
Partnered	80.7	0	100	76.0	0	100
Married	62.8	0	100	57.4	0	100
Cohabiting	18.0	0	100	18.6	0	100
Single	19.3	0	100	24.0	0	100
Home owner	75.7	0	100	72.8	0	100
Missing info net income	5.0	0	100	5.3	0	100
College	34.0	0	100	26.8	0	100
Drinking	73.4	0	100	56.1	0	100
Smoking	21.3	0	100	18.4	0	100
Missing info physical problem	5.2	0	100	5.0	0	100
Different-sex	74.1	0	100	69.5	0	100
Same-sex	1.4	0	100	1.7	0	100
Unknown orientation	24.5	0	100	28.8	0	100
Age to 19	4.3	0	100	5.2	0	100
Age 20 to 29	8.3	0	100	10.6	0	100
Age 30 to 39	12.8	0	100	15.2	0	100
Age 40 to 49	17.9	0	100	18.9	0	100
Age 50 to 59	20.5	0	100	21.1	0	100
Age 60 to 69	23.0	0	100	18.8	0	100
Age 70 plus	13.2	0	100	10.2	0	100

Based on 12,955 observations of 3,088 men and 14,824 observations of 3,617 women.

Table S3: Parameter Estimates Effects of Partnership on Subjective Well-being; Full Baseline Model

	Males		Females	
Marriage	0.33	(0.08)**	0.39	(0.08)**
Cohabitation	0.21	(0.07)**	0.21	(0.07)**
Children number	-0.04	(0.03)	-0.07	(0.03)**
Home owner	-0.08	(0.07)	-0.02	(0.06)
Log(net income)	0.04	(0.01)**	-0.00	(0.01)
Missing info net income	0.30	(0.12)*	-0.19	(0.09)*
College	0.09	(0.08)	0.10	(0.08)
BMI	0.01	(0.01)	-0.00	(0.00)
Physical problem	-0.03	(0.01)*	-0.02	(0.01)
Missing info physical problem	-0.02	(0.04)	-0.00	(0.04)
Smoking	0.09	(0.04)†	0.04	(0.05)
Drinking	-0.02	(0.03)	0.03	(0.03)
Drinking days	-0.00	(0.01)	-0.01	(0.01)
Age 20 to 29	-0.13	(0.08)†	-0.11	(0.07)†
Age 30 to 39	-0.34	(0.12)**	-0.14	(0.10)
Age 40 to 49	-0.45	(0.13)**	-0.15	(0.11)
Age 50 to 59	-0.56	(0.14)**	-0.13	(0.12)
Age 60 to 69	-0.44	(0.15)**	-0.09	(0.13)
Age 70 plus	-0.39	(0.16)*	0.05	(0.15)
Constant	7.37	(0.21)**	7.62	(0.16)**

12,955 observations of 3,088 men; 14,824 observations of 3,617 women

Standard errors in parentheses; † $p < 0.1$; * $p < 0.05$; ** $p < 0.01$
